# Retinopathy of prematurity in India – what can we learn from the polio legacy?

**DOI:** 10.1016/j.lansea.2023.100210

**Published:** 2023-05-10

**Authors:** Sam Ebenezer Athikarisamy, Anand Vinekar, Sanjay Patole

**Affiliations:** a Neonatal Directorate, Child Adolescent Health Service, Perth, Western Australia, Australia; b School of Medicine, University of Western Australia, Crawley, Western Australia, Australia; c Director, KIDROP, Narayana Nethralaya Eye Institute, Bangalore, India

**Keywords:** Retinopathy of prematurity, Global polio eradication initiative, Screening, Surveillance, Key performance indicators

## Abstract

Retinopathy of prematurity (ROP) is a vasoproliferative disease of the preterm retina that has the potential to cause vision impairment and blindness. Timely screening and treatment are hence critical for infants at risk for ROP. Screening for ROP is challenging in India owing to the limited resources, a vast at-risk population and lack of awareness among paediatricians and the public. Addressing ROP in India requires a comprehensive approach involving multiple sectors, considering the magnitude of the problem and the expected increase in need for ROP services due to the increased survival of preterm infants following improvements in neonatal care. The success of the Global Polio Eradication Initiative (GPEI) offers valuable lessons for improving ROP services in developing nations by applying its strategies. An approach for primary and secondary prevention of ROP is proposed, and the current challenges and a neonatal-led care model for ROP are discussed.

## Background

Retinopathy of prematurity (ROP) is a vasoproliferative disease affecting the retinal vessels of premature infants and can lead to severe vision impairment if not identified and treated at the right time.^1^ The survival of preterm infants has significantly increased globally in the last two decades, especially in countries such as India, with over 3.5 million preterm infants born and surviving annually.^2^ The occurrence of ROP in India and other developing nations, also known as the “third epidemic” of ROP, is the result of a combination of uncontrolled supplemental oxygen (first epidemic pattern) and the evolving but uneven care of very preterm infants (second epidemic pattern).^3^^,^^4^ Blindness in infancy can lead to many disability-adjusted life-years lost^5^ and is considered a developmental emergency.

Thirty years ago, the prevalent form of disability in developing nations was residual paralysis caused by wild poliovirus, resulting in over 350,000 annual cases of paralysis, primarily involving children, and was endemic in 125 countries.^6^^,^^7^ In response, the World Health Assembly passed a resolution to eradicate polio by the year 2000, leading to the creation of the Global Polio Eradication Initiative (GPEI), the largest partnership between the public and private health sectors.^6^^,^^7^ Today, the world is close to eliminating polio.^8^ In the post-polio era, untreated ROP poses a significant risk for severe and irreversible visual impairment. On the disability scale, blindness ranks high in severity and has a significant impact on a child’s quality of life and global development.^5^^,^^9^^,^^10^

Blindness in childhood can have long-lasting consequences for the affected child and family, profoundly impacting educational, employment, personal, and social prospects. The effects of childhood blindness can be more severe than those of adult-onset blindness.^9^^,^^10^ A cost of illness study (2022) in India using gross national income (GNI), disability weights, and productivity metrics estimated the economic burden of blindness and visual impairment as a net loss of 845 billion Indian rupee (INR) in GNI due to blindness and a per capita loss of 170,624 INR in GNI per blind person.^11^

An estimated 32,200 cases of blindness and visual impairment worldwide were attributed to ROP in 2010, with the greatest burden observed in middle-income countries. India alone accounts for ∼10% of the global estimate, with an estimated 5000 children developing severe ROP and 2900 surviving with visual impairment.^12^ A study evaluating the trend from 2000 to 2017 from a tertiary centre in South India observed a 20-fold increase in the number of children diagnosed with ROP and a 12-fold increase in those needing treatment.^13^ Limited epidemiological data are available on blindness caused by ROP in India. However, it is important to note the recent increase in studies reporting on surgical procedures and outcomes for stage 4 and 5 ROP from various regions of India. The reasons cited for the advanced stages of the disease include late referral and poor follow-up practices.^14^, ^15^, ^16^

The success of the GPEI shows that India can provide health care to every child in the country irrespective of how marginalized and remotely based they are. The tangible assets of GPEI (e.g., laboratory network, global surveillance) may not be relevant to ROP. However, intangible assets such as the knowledge, experience, process, system, and activities learned from the initiative will be invaluable for ROP control. The lessons learned from GPEI include understanding the importance of ‘(1) mobilizing political and social support, (2) strategic planning and policy development, (3) partnership management and donor coordination, (4) program operations and tactics, and (5) oversight and independent monitoring’.^6^

We discuss how the strategies responsible for the success of GPEI can be used to control ROP-related blindness in India while acknowledging that the diagnosis and treatment of ROP may require more specialised resources compared to polio. An approach for primary and secondary prevention of ROP is proposed, and the current challenges and a neonatal-led care model for ROP are discussed.

## Learning from GPEI

### Mobilizing political and social support

Managing a serious public health issue such as ROP on a national level requires strong societal and political commitment. “VISION 2020: the Right to Sight,” a joint initiative of the World Health Organization (WHO) and the International Agency for the Prevention of Blindness (IAPB), was established in 1999 with the aim of eliminating avoidable blindness by 2020 and preventing the projected doubling of avoidable visual impairment from 1990–2020.^17^^,^^18^ The strategies for achieving these goals included ‘a comprehensive, high-quality, equitable eye care system integrated into national healthcare systems’, with a focus on treating ROP as one of the treatable causes of childhood blindness. The target was to ensure that all at-risk infants received fundus examination by a trained observer. Despite significant advancements, the COVID-19 pandemic has overshadowed the achievements of “VISION 2020,” and there is still much work to be done to reach its goals. A report published in The Lancet examined the lessons learned from “VISION 2020” and proposed a comprehensive plan to address future needs, emphasizing the importance of the eye health workforce and the need for expanding the service capacity through increased numbers, improved training, better working conditions, and effective leadership.^19^

Nongovernmental organizations (NGOs), such as the ‘Public Health Foundation of India’ through Queen’s Diamond Jubilee Trust (2012), London, UK, worked in partnership with state governments to address this issue.^20^ Over the last decade, there have been multiple partnerships that have helped establish a screening model in the states of Maharashtra and Odisha.^21^^,^^22^

Paediatricians, neonatologists, and neonatal nurses play a significant role in the prevention and monitoring of ROP, as well as their role in monitoring cases of acute flaccid paralysis (AFP) following polio.^23^ Obstetricians have a key role in reducing the occurrence of ROP by addressing maternal health factors that contribute to the development of the condition. This includes promoting healthy diets, managing hypertension, reducing obesity, and preventing preterm births.^24^

The Polio program was greatly benefited by brand ambassadors who helped galvanize nationwide awareness about the disease. Film stars, sportsmen and publicly recognized personalities contributed to the short but effective public messages carried out in mass, print and social media at regular intervals. Such an effort is required for the ROP program.^25^ The success of the GPEI in engaging political leaders and implementing accountability frameworks can be applied to ROP programs in India, particularly at the state and district levels.^26^

### Strategic planning and policy development

The need for strategic planning and policy development in India has been emphasized by many since 1990.^27^ Strategic planning and policy development was a critical component of GPEI.

Jalali et al. (2003) proposed a model for screening and timely intervention to prevent blindness due to ROP.^28^ In 2010, the National Neonatology Forum in India and ophthalmologists released guidelines for screening preterm infants for ROP.^29^ In 2015, the Ministry of Health, India integrated ROP screening into the Rashtriya Bal Swasthya Karyakram (RBSK) and the National Program for Control of Blindness (NPCB).^30^

In 2017, the National Task Force of ROP in India issued operational guidelines for ROP.^31^ These guidelines suggest that preterm infants born at <34 weeks of gestation OR with birth weights under 2000 g (if gestation at birth not known) should be screened for ROP and screen larger infants born between 34 and 36 weeks if they have high-risk factors. The first screening is recommended to occur within 4 weeks of life, and infants with birth weight <1200 g or gestation <28 weeks should be screened at 2–3 weeks of life.^32^ Operational guidelines were developed to assist in the expansion of these services in both public and private healthcare sectors. However, the adoption of these guidelines in clinical practice across India has not been optimal.^33^^,^^34^

### Public private partnership (PPP)

The Indian National Health Policy has set a goal of raising healthcare spending to 2.5% of GDP by 2025 and sees PPP to improve healthcare delivery and bring in private sector resources.^35^ PPPs have been successful in India's healthcare sector, making medical care accessible to all social classes. Similar to the GPEI, there is a need for PPP and interagency coordination between all stakeholders to mobilize resources and increase advocacy for ROP. The WHO, UNICEF, Centers for Disease Control and Prevention, Rotary International, and the Bill & Melinda Gates Foundation formed an exceptional and dedicated global partnership to support the GPEI and worked relentlessly to overcome the challenges.^36^ A similar approach is needed to address ROP.

Until the government implements a nationwide universal ROP screening program, it should seek the support of nongovernmental and semigovernmental organizations for ROP screening. These partners can contribute to the national program and test different ROP screening models in real-world settings, which can later be adopted and sustained by the government. The examples mentioned below are successful models of PPPs in providing ROP screening and treatment.

The Karnataka Internet-assisted Diagnosis of ROP (KIDROP) model is a tele-ROP service developed in Bangalore, Karnataka, India that uses nonphysician imaging staff to capture images to provide ROP screening and subsequent treatment in geographic regions lacking ROP specialists. Trained imaging staff capture and interpret retinal images using a triaging algorithm, and ophthalmologists from the centre view images in near real-time on their smartphones to provide reports and management decisions within minutes. The KIDROP model has enabled families in rural areas to access ROP specialists remotely, improving access to care and potentially preventing blindness in infants with ROP.^37^ This successful model has been implemented state-wide and has provided training and mentoring to institutions across the country and internationally, leading to the development of similar programs.^38^ An economic impact study found that the implementation of this model in other states with similar demographics could potentially save over 100 million USD annually in blindness-related costs.^39^

‘Retinopathy of Prematurity Eradication Save Our Sight (ROPE-SOS)’–a Tele-screening Project is another successful example of a PPP in providing ROP screening and treatment and providing services for babies in the Western districts of Tamil Nadu, Northern Districts in Kerala in South India.^40^

The Queen Elizabeth Diamond Jubilee Trust played a crucial advocacy role in partnering with the government and public health institutions to organize ROP symposiums, establish a national task force and technical expert groups, and launch ROP screening programs in four states using an ophthalmologist model by training 22 ophthalmologists to screen and 9 ophthalmologists to treat ROP.^41^^,^^42^

### Program operations and tactics

The extensive surveillance system consisting of people, communication technology, transport, and data management that was instrumental in the success of the GPEI is crucial for managing ROP programs.

**Fig. 1:**
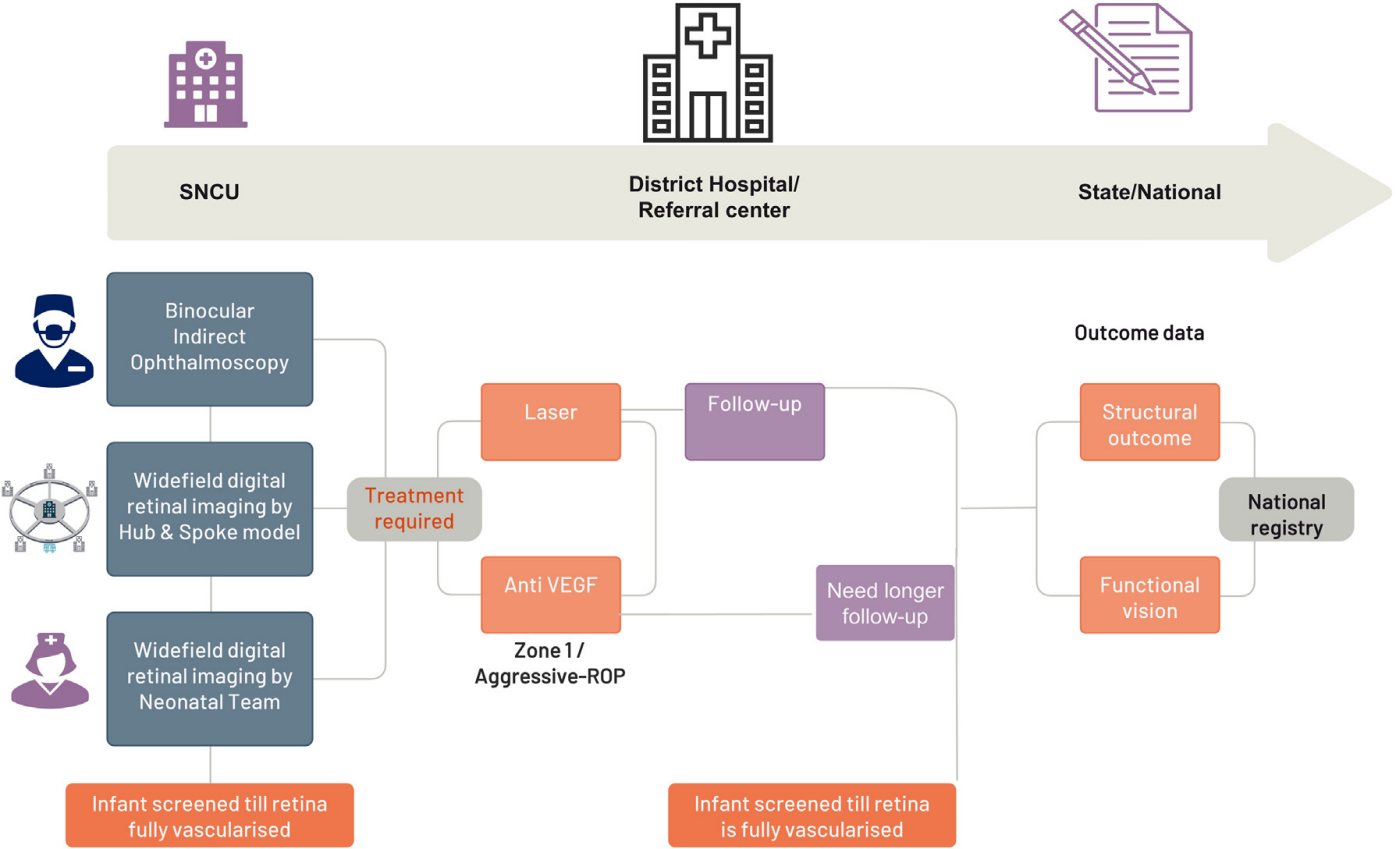
**An overview of retinopathy of prematurity surveillance with regular reporting.** To improve the accuracy of ROP incidence rates and facilitate timely interventions for at-risk infants, ROP screening programs could adopt a polio surveillance-style model that requires regular reporting of every eligible infant’s ROP screening outcome, including submitting regular ‘zero reports’ when no ROP cases are detected.

In the polio surveillance program, health facilities were required to report all cases of AFP and submit weekly ‘zero reports’, even when no AFP cases were detected.^43^ This practice helped to ensure accurate reporting and could also be applied to ROP screening programs in India to encourage regular reporting of all ROP cases and zero cases (Fig. 1). Similar to the GPEI, it is important to adapt and optimize strategies for ROP programs based on ongoing research and data analysis. The development and implementation of new technologies, such as tele-imaging, improved screening methods, and evidence-based options, can play a significant role in the success of ROP programs. Operational research is essential to ensure that interventions are effectively reaching the target population. Special strategies may be needed to reach underserved and migrant populations. With advancements in neonatology, neonatal intensive care units (NICUs) are now located not only in capital cities but also at the district and taluk head quarter levels.^44^ Hence, developing strategies to reach underserved rural and remote areas is important. The success of the Social Mobilization Network (SMNet) in promoting polio vaccination in India could serve as a model for raising awareness and advocating for ROP screening and treatment. Engaging frontline social mobilizers and utilizing targeted communication and outreach could increase awareness and participation in ROP programs, particularly in underserved and at-risk communities.^45^

Like the AFP surveillance system, conducting sensitization sessions and awareness seminars on ROP screening for clinicians and other healthcare workers, tracking and counselling pregnant mothers during antenatal visits, enhancing the interpersonal skills and motivation of healthcare workers, and investing in their education and training can improve the success of ROP screening and treatment. Creating a uniform accreditation for imaging staff and providing online training modules (self-assessments, video sessions), such as WISE-ROP, can address the limitation of tele-ROP programs and ensure trained and compliant imagers for ROP screening.^38^ Targeted communication and outreach, advocacy for ROP service improvements, and tracking and follow-up of high-risk infants are crucial for effective ROP programs.^46^^,^^47^ As part of the Queen Elizabeth Trust’s pilot ROP screening program in four states, a software platform was established for online data collection and monitoring to facilitate tracking of infants in need of screening and treatment.^41^

### Oversight and independent monitoring

#### Independent monitoring board (IMB)

The GPEI established an IMB to monitor progress and guide the effort towards ending polio transmission globally.^48^ The IMB met regularly with program officials and provided recommendations on individual countries and global program issues, playing a key role in elevating the priority of polio eradication, emphasizing the importance of human factors, encouraging innovation, focusing on polio sanctuaries, and continuously improving program quality. The IMB’s independence allowed it to address difficult issues that others cannot, making it an asset for the GPEI.^48^

The principles and characteristics of the strong oversight and independent monitoring implemented in the GPEI can be applied to ROP programs. These include the formation of an IMB to assess progress towards the prevention of ROP and the establishment of a central technical advisory body to provide ongoing guidance and direction with regards to prevention of preterm birth and safe use of oxygen. The involvement of other international organizations can help secure commitment and support from member states for ROP programs.

The Indian ROP (IROP) society was established in 2016 to prevent blindness in infants with ROP by promoting standards of excellence. Its objectives include promoting collaboration among ROP specialists and interacting with other healthcare providers, such as paediatricians and obstetricians, highlighting best practices, adhering to national guidelines, promoting ethical standards, encouraging technological advances, developing low-cost technological advances, collaborating for trials, liaising with the government to integrate ROP care with national goals, and safeguarding medico-legal interests.^49^

The IROP Society can establish an independent monitoring mechanism similar to GPEI to ensure that ROP care providers adhere to national guidelines and provide ethical and effective treatment to at-risk infants. This is particularly important given the increasing demand and rapid progress in the field of ROP with new treatment options and technological advancements. The society can audit and accredit ROP care providers, create a directory of certified professionals, and standardize the training curriculum.

To set target milestones and standard performance indicators and monitor progress, the IROP society can collect data on ROP screening and treatment outcomes through a standardized reporting system. Promoting clinical and operational research can help develop a comprehensive ROP screening and surveillance model. Engaging with community organizations and media outlets in an innovative way will be crucial to raise awareness about ROP. Society can collaborate with the central and state governments to advocate for increased funding and engage with stakeholders to ensure that ROP care is integrated into existing health systems.

A survey (2021) of the practice patterns of its members in relation to anti-vascular endothelial growth factor (anti-VEGF) therapy is an example of the steps the IROP Society can take towards achieving its goals.^50^ Society can also play a pivotal role in evolving the combined guidelines and preferred practice patterns by engaging with experts and stakeholders in the field. The IROP Society’s work in India has the potential to serve as a model for other countries facing similar ROP epidemics.^51^

#### Performance indicators

**Fig. 2:**
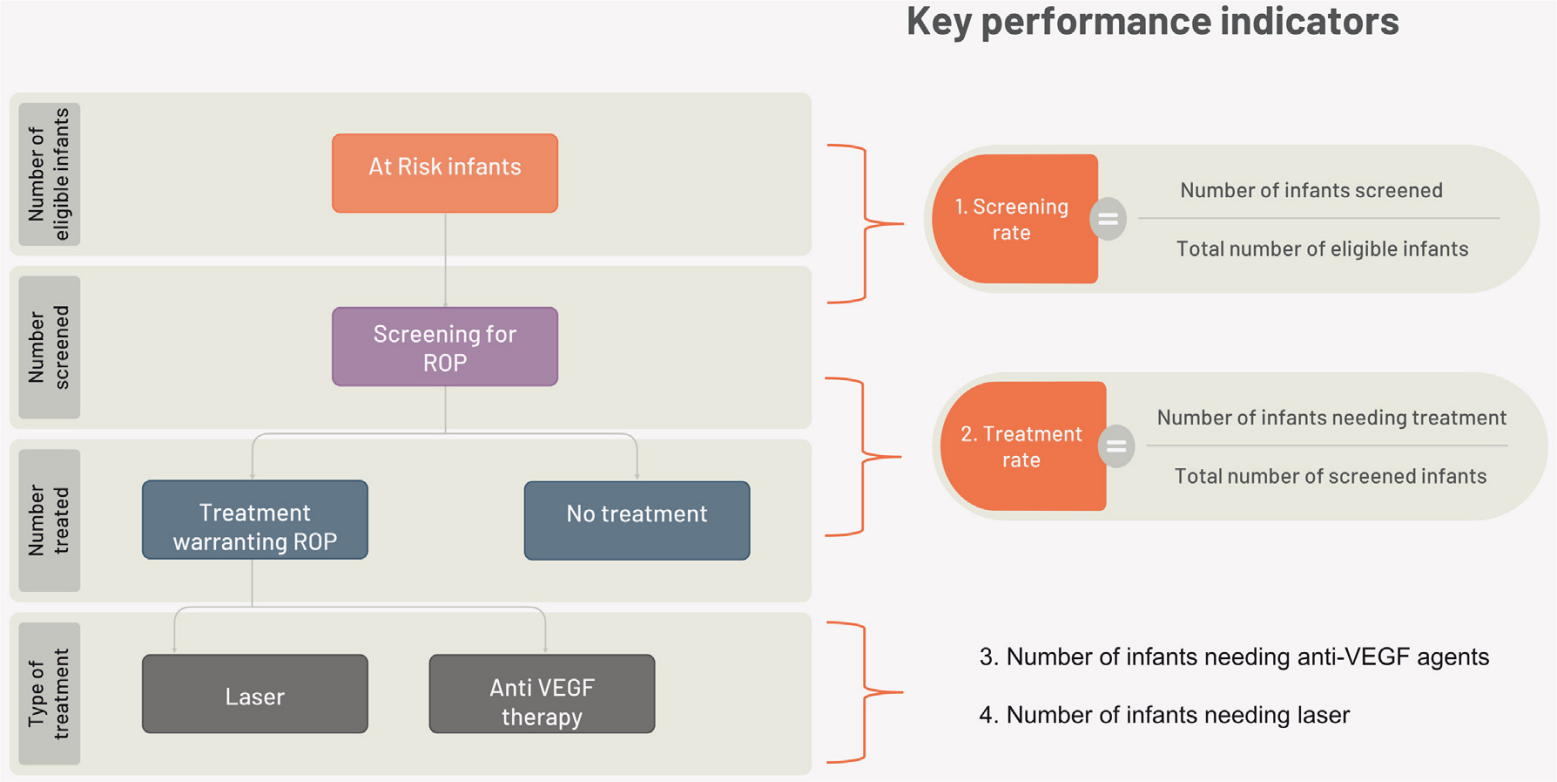
**Key performance indicators related to retinopathy of prematurity surveillance**.

The Eye Care Indicator Menu (ECIM), a set of eye care indicators selected by the WHO, can be used to monitor and evaluate national eye care plans, identify gaps and successes, and advocate for resource allocation. Among the indicators is indicator 12, which measures the coverage of ROP screening in eligible preterm infants. The numerator counts the number of eligible infants screened, and the denominator represents the total eligible infants admitted to neonatal care (Fig. 2). The indicator can be broken down by sex, geography, sector, and socioeconomic status and is measured annually using routine health facility data. The ECIM is a valuable resource for developing or enhancing an eye care monitoring framework that integrates into the broader health monitoring and evaluation framework.^52^

The use of surveillance and program performance indicators can also be applied to objectively monitor national and global progress in the prevention and treatment of ROP. Performance indicators will be important for benchmarking at the state, national, and international levels. Evaluation is a critical component of program management, as it provides valuable information on the effectiveness and impact of a program. The evaluation of the KIDROP program using the CDC guidelines in 2015 is one such example where the report helped in demonstrating the impact of the program on all stakeholders.^37^^,^^38^

## Prevention of ROP

Having discussed the components of the GPEI and their use in ROP surveillance, it is important to consider the role of primary and secondary prevention in the context of ROP. The GPEI has demonstrated the importance of a comprehensive approach to disease eradication, including measures for primary and secondary prevention. In the case of ROP, strategies for primary prevention aim to reduce the incidence of the condition, whereas strategies for secondary prevention aim to detect and treat the condition in its early stages to prevent vision loss. We discuss these strategies and their integration into existing surveillance programs for ROP.

### Primary prevention

#### Reducing preterm birth

Preterm birth (PTB), which accounts for 12% of pregnancies worldwide, is a leading cause of neonatal morbidity and mortality.^53^^,^^54^ Although there are only a limited number of interventions available to prevent PTB, prophylactic administration with antenatal corticosteroids (ACS) in those who meet the conditions for use has improved neonatal outcomes. However, the use of ACS and antibiotics has not reduced the incidence of PTB. Prophylactic administration of progesterone to women with a history of preterm delivery and those identified with a short cervical length using routine transvaginal ultrasound may be effective in reducing the rate of PTB.^54^^,^^55^

A cross-sectional study conducted in Haryana, India, assessed the readiness of 37 public health facilities to provide ACS in accordance with the 2014 operational guidelines released by the Indian government.^2^ The study found that while the guidelines for ACS use were in place, dissemination of these guidelines was suboptimal with poor implementation. Improved supervision and standardization of threatened preterm birth care are needed to ensure safe and effective ACS use. The authors suggest updating guidelines to include specific actions based on recent scientific evidence.^2^ The recently published WHO ACTION trial, which included women from India, provides convincing evidence for ACS to reduce the complications associated with preterm birth in LMICs when implemented in adequately resourced health settings.^56^

#### Oxygen stewardship

Prevention of prematurity, the single most important risk factor for ROP, is difficult. While the pathogenesis of ROP is multifactorial, the use of oxygen is a critical factor in its development, especially in preterm infants.^57^ The increase in preterm survivors due to the rapid but uneven progress of neonatal intensive care is one of the main reasons for the rising burden of ROP in India. The other significant contributory factors are the lack of awareness of ROP among paediatricians and the unavailability of oxygen blenders and saturation monitors.^58^

A recent survey from four special care neonatal units found that only one out of 18 neonates receiving oxygen had an accurately set upper oxygen saturation alarm, and none of them were monitored continuously. The majority (84%) of nurses were not aware of the ideal oxygen saturation targets.^59^ A recent analysis of retinal images from Indian NICUs showed that a significant proportion (75%) of larger preterm infants who were exposed to 100% oxygen experienced vascularization loss. These infants were relatively more mature (mean gestation: 31.7 weeks) and heavier at birth (mean birthweight: 1572 g).^60^

The WHO recently published standards for improving care for small and sick newborns.^61^ The guidelines recommend the use of neonatal pulse oximeters to guide the administration of supplemental oxygen and safe delivery of oxygen through appropriate neonatal equipment. Preterm newborns born before 32 weeks of gestation who require respiratory support should receive oxygen within a range of 21%–30%. It is important to regularly review oxygen concentrations to maintain a target oxygen saturation range of 90%–95%. These recommendations are outlined in the WHO Quality statement (1.17–1.19).^61^

Promoting safe oxygen delivery and optimizing saturation targets through safety protocols for paediatricians can have a significant impact on the incidence and health burden of ROP. Recent quality improvement efforts have demonstrated success in decreasing the unindicated use of oxygen. The implementation of a targeted ‘oxygen policy’ using quality improvement principles in a level II neonatal unit in Madhya Pradesh reduced the consumption of oxygen cylinders by 63% and decreased the annual maintenance budget by over 40%. The policy was based on the assumptions that adopting WHO-recommended low-flow nasal prongs and implementing clear guidelines for oxygen use would improve efficiency and reduce costs with an estimated saving of 5000–7000 L of oxygen/neonate/day. The policy comprised two key interventions: a written ‘oxygen policy’ outlining indications for starting/stopping oxygen and saturation targets and using short binasal infant prongs at 0.5–1 L/min instead of oxygen hoods for oxygen delivery in spontaneously breathing neonates.^42^^,^^62^ The modelling of neonatal care in the future should prioritize safe delivery of oxygen through blenders and monitoring equipment, as well as implementation of an inbuilt referral system for ROP screening. Advocates have recommended that these features be included as part of the commissioning process.

#### Comprehensive care for preterm infants

It is important to note that sepsis, poor nutrition, and exposure to blood products are other modifiable factors involved in the development of ROP.^63^, ^64^, ^65^ The ‘Preterm New-born Health Care Package’ (PHCP) has been developed jointly by the WHO Collaborating Centre, All-India Institute of Medical Sciences, and the Queen Elizabeth Diamond Jubilee Trust for improving facility-based care of preterm infants in India. It includes strategies such as improved oxygen administration, use of continuous positive airway pressure and surfactant, reduced blood sampling, early feeding with breast milk, aseptic techniques, prevention of hypothermia, kangaroo mother care, pain control, and increased parental involvement. The educational package is an important tool for the ongoing dissemination of knowledge and promotion of best practices in neonatal care.^66^

Preterm human milk, rich in antioxidants and growth factors (e.g., insulin-like growth factor), can help in the prevention of ROP.^67^ A meta-analysis of 5 studies found that feeding with human milk instead of formula significantly reduced the odds of developing any ROP (OR: 0.31, 95% CI: 0.19–0.49, P < 0.001).^68^ A recent survey by Thuileiphy et al. (2022) highlights the need for educating mothers of preterm infants to improve their knowledge and attitude towards breastfeeding.^69^

### Secondary prevention

#### ROP screening

A survey (2011) of 241 paediatricians from six states in India showed that at least one-third (34%) of them were not referring patients for ROP screening.^70^ Data (2013–2017) from Chandigarh showed that nearly 15.6% of infants who presented with stage 4B/5 ROP were due to delayed or no screening.^71^ A retrospective study by Padhi et al. found that late presentation (71 infants) of ROP in a tertiary eye care institute in Eastern India was due to system and neonatal care policy failure in 63.3% of cases, parental ignorance in 26.7% and ophthalmologist misdiagnosis/unavailability in 10% of infants. Most of the infants (63.3%) were admitted to the NICU past the due date for ROP screening and had an average stay of 35.5 days.^72^ A questionnaire-based survey from Palakkad district, Kerala, showed that paediatricians and general practitioners in the district were aware of ROP and the need for screening. However, only 23.33% of general practitioners and 60.82% of paediatricians were aware of the appropriate timing for screening.^73^ Another survey among NICU nurses from a tertiary unit found a significant knowledge gap related to ROP and its prevention strategies. Using quality improvement methods, healthcare providers were able to improve parental awareness, nurse knowledge, and ROP screening rates in a short time frame without needing additional resources or manpower.^74^ Another quality improvement initiative increased ROP screening rates from 10.7% in the preintervention phase to 87.3% in the postintervention phase in a tertiary NICU in northern India.^75^

ROP screening is crucial to prevent blindness, and different screening models are available. In Table 1, we provide a description of the binocular indirect ophthalmoscopy (BIO) model by ophthalmologists,^78^ tele-imaging model using wide-field digital retinal imaging (WFDRI) (a hub and spoke model),^76^ and the tele-imaging model using WFDRI by healthcare workers in respective units (neonatology led model).^77^

**Table 1:** Screening models for the low-middle-income countries.

	Binocular indirect ophthalmoscopy by ophthalmologists	Tele-imaging^37^^,^^40^^,^^76^ hub and spoke model	Neonatology lead model^77^
Team	Ophthalmologists work in their respective SNCUs, and they are linked with the nodal centre	Imaging technicians and program co-ordinator work alongside remotely located Ophthalmologist	NICU nurses/paediatricians work alongside remotely located Ophthalmologist
Program coverage	Only to their respective SNCUs	One team can cover up to 5–6 districts in the current model	Respective SNCU/NICU
Technique	Direct examination of the retina using Binocular Indirect Ophthalmoscopy	Fundus images are taken by certified technicians	Fundus images are taken by certified nursing staff/doctors
Training	Done by qualified paediatric ophthalmologists	Level 1–3 technicians	Neonatal nursing or medical staff
Pros	Cost effective	Possible to screen large number of at-risk infants and possible to integrate with AI in the future	‘Neonatal team owns the responsibility’ to screen all eligible infants and possible to integrate with AI in the future
Cons	There is a shortage of ophthalmologists trained to manage ROP and to meet the demand. Private NICU’s may not be able to access the service. No stored images for longitudinal viewing.	Requirement for an administrative framework, including credentialing, to safeguard the imaging staff	Each neonatal unit requires a designated leader, imaging staff and imaging camera. Need high level of multidisciplinary coordination.

SNCU; special newborn care unit, NICU, neonatal intensive care unit, AI: artificial intelligence.

#### ROP treatment

Infants who meet the criteria for treatment as per the ETROP^79^ guidelines should be managed appropriately with peripheral retina ablation by laser in their respective units or tertiary care centres or nearby teaching hospitals. A proper referral system should be in place for hospitals that do not have an inhouse facility for laser treatment. Intravitreal anti-VEGF injections have become an alternative treatment option for ROP, particularly for Zone 1 disease. Compared to laser treatment, easy access to anti-VEGF drugs may lead to treatment decisions being based on availability rather than clinical indication, emphasizing the importance of using these drugs cautiously and considering the need for longer follow-up.^80^

The recent survey by Gangwe et al. that showed an increasing use of anti-VEGF agents for severe ROP and significant variations in practice regarding drug selection, dosage, monitoring for reactivation, and documentation highlights the need for standard guidelines. It is important to note that the need for close and prolonged monitoring in infants receiving anti-VEGF agents for ROP poses a significant challenge, especially in low- and middle-income nations.^50^

### Long-term monitoring for visual problems

Monitoring for long-term visual issues is important in preterm infants with ROP.^81^ Complications such as myopia, hypermetropia, astigmatism, anisometropia, and strabismus can cause permanent visual impairment if not addressed in a timely manner.^81^^,^^82^ Cryotherapy for ROP (CRYO-ROP) and Early Treatment for ROP (ETROP) studies have reported the long-term refractive consequences of ROP.^83^^,^^84^ Significant refractive errors are more common in infants with severe ROP than in those with no or mild ROP.^85^ Studies comparing intravitreal anti-VEGF agents (e.g., bevacizumab and ranibizumab) with conventional lasers have shown that refractive errors are more common after laser treatment.^86^^,^^87^ Overall, these data emphasize the importance of continual monitoring and timely intervention for vision problems in infants with significant ROP. The operational guidelines recommend that infants who require treatment should be followed up until at least 5 years of age.^31^

### Role of artificial intelligence in ROP screening

Campbell et al. evaluated the effectiveness of an Indian telemedicine program for ROP that uses artificial intelligence (AI) for screening and assessed whether AI-identified differences in ROP severity between neonatal units are related to differences in oxygen-titrating capability. They found that the severity of ROP based on the ROP severity score was higher in NICUs without oxygen blenders and pulse oximeters. Thus, AI has the potential to be integrated into ROP screening programs and used for monitoring disease surveillance in different NICUs.^88^

Integrating AI technology with wide-field imaging (WFDI) offers promising prospects for the future of ROP screening in India. Reliable AI systems for ROP screening are currently being investigated by several groups in India.^89^^,^^90^ WFDI can provide a sustainable and scalable model for ROP screening by non-ophthalmologists. Furthermore, AI can improve interdisciplinary coordination in ROP screening. However, it is important to note that AI is still in the early phase of development.^89^

### Current challenges

ROP screening and management in India face significant challenges that extend beyond the shortage of the screening workforce. These include a lack of uniformity in ROP screening across the nation, often due to a lack of resources. There is limited collaboration between paediatricians and ophthalmologists and suboptimal coordination between the public and private sectors. With the increasing use of anti-VEGF agents for ROP treatment in India, there is a pressing need to monitor long-term effects, including both ocular and neurological outcomes, after such an intervention. Inadequate facilities for neurodevelopmental follow-up of high-risk infants are a significant challenge in India, unlike in high-income countries, where such programs are well integrated into the healthcare system. Addressing these multifaceted challenges requires collaboration between healthcare professionals, policymakers, and stakeholders to ensure optimal care and outcomes for preterm infants at risk of ROP.^49^

The recent updates to the ICROP guidelines,^91^ which aim to improve the objectivity of findings and incorporate clinical variations in ROP regression and reactivation, can present difficulties at the community level. This is particularly relevant after treatment with anti-VEGF. Hence, there is a need for mentoring and supervision from larger nearby centres to ensure effective management.

**Fig. 3:**
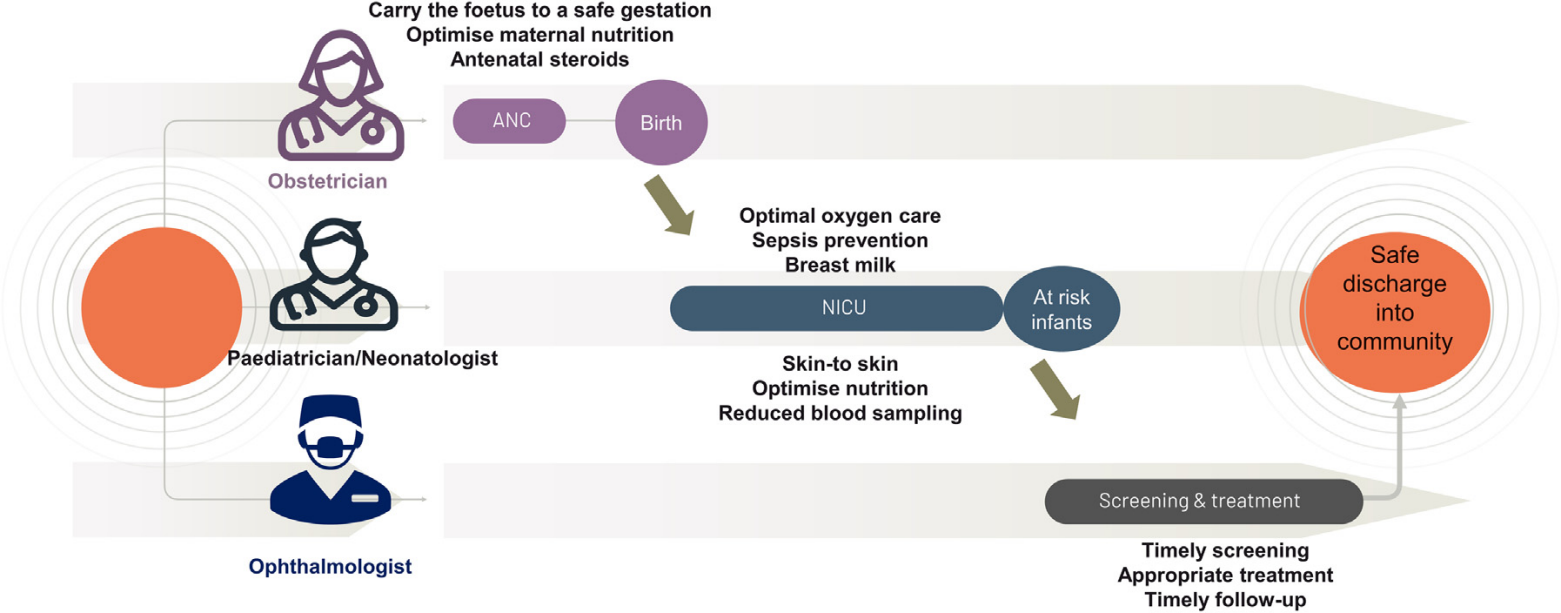
**A schematic representation of the process of preventing retinopathy of prematurity using a relay race analogy.** Each subspecialist takes over from the previous one, similar to how a swimmer hand over the baton to their teammate in a relay race. The obstetrician initiates the process in the antenatal period by optimizing fetal development and prolonging gestation. The paediatrician takes over by identifying and reducing risk factors, screening (inhouse) in some instances and referring at-risk infants to ophthalmologists. Finally, the ophthalmologist takes over the baton, devising and implementing the treatment plan and providing ongoing follow-up care. Collaboration and communication between subspecialists are critical for the successful prevention and management of ROP.

Despite the existence of established guidelines and standards for ACS administration, oxygen administration and saturation targeting, and screening for ROP, adherence to these guidelines by healthcare providers remains suboptimal.^34^^,^^59^ Effective measures are required to promote adherence to established guidelines for ROP management. Focusing on addressing attitudinal barriers among healthcare providers can facilitate the uptake of best practices in ROP management, ultimately improving patient outcomes. Academic bodies, such as the Indian Academy of Pediatrics and the National Neonatology Forum, can play a critical role in advocating the adoption of established guidelines for ROP management among obstetricians, pediatricians and neonatologists.^92^^,^^93^ To mitigate the risk of malpractice litigation in ROP, it is important to update and comply with the guidelines and maintain the standard of care - when dealing with high-risk infants.^94^ Coordination among hospital staff (obstetricians, paediatricians and ophthalmologists) and parents is essential to ensure timely screening (Fig. 3).

### A future ‘neonatal-led’ model for ROP screening

The vision for ROP screening is a universal and standardized screening program that utilizes innovative technologies. The ideal screening programme would be a collaborative effort led by paediatricians and neonatologists at the unit level, leveraging telemedicine, low-cost infant imaging cameras, and possibly AI.^95^ By employing a cadre of trained technicians and an accredited reading centre, remote screening sites can receive rapid and credible diagnosis, leading to timely interventions to prevent severe vision loss and blindness. With a concerted team effort and continued innovation, this vision for ROP screening can become a reality, providing optimal care and outcomes for all premature infants at risk of ROP.^95^

## Conclusion

Learning from past public health campaigns such as the GPEI can inform the design of effective interventions for ROP. Evidence suggests that the WFDI approach may be more suitable for addressing ROP in community settings with a limited trained workforce and vast geographical area. Experience from countries such as Costa Rica and Cuba suggests that mandatory legislation for ROP screening may be unnecessary if the government supports ROP programs, develops national guidelines, and establishes data collection and monitoring systems.^96^ Although elimination of ROP may not be feasible according to the Dahlem principles of disease, adopting an evidence-based approach to management can still effectively prevent blindness due to ROP.^97^ The cost of implementing ROP screening programs is outweighed by the cost of inaction.

## Contributors

S A, A V and S P conceptualized the idea and developed the outline of the article. S A provided the first draft of the manuscript. All authors contributed to the writing and finalization of the manuscript.

## Declaration of interests

We declare no competing interests.
